# A Rapid Genetic Assay for the Identification of the Most Common *Pocillopora damicornis* Genetic Lineages on the Great Barrier Reef

**DOI:** 10.1371/journal.pone.0058447

**Published:** 2013-03-07

**Authors:** Gergely Torda, Sebastian Schmidt-Roach, Lesa M. Peplow, Petra Lundgren, Madeleine J. H. van Oppen

**Affiliations:** 1 School of Marine and Tropical Biology, James Cook University, Townsville, Queensland, Australia; 2 Australian Institute of Marine Science, Townsville, Queensland, Australia; 3 Australian Research Council Centre of Excellence for Coral Reef Studies, James Cook University, Townsville, Queensland, Australia; 4 AIMS@JCU, James Cook University, Townsville, Queensland, Australia; 5 Institute for Antarctic and Southern Ocean Studies, University of Tasmania, Hobart, Tasmania, Australia; 6 Great Barrier Reef Marine Park Authority, Townsville, Queensland, Australia; University of New South Wales, Australia

## Abstract

*Pocillopora damicornis* (Linnaeus, 1758; Scleractinia, Pocilloporidae) has recently been found to comprise at least five distinct genetic lineages in Eastern Australia, some of which likely represent cryptic species. Due to similar and plastic gross morphology of these lineages, field identification is often difficult. Here we present a quick, cost effective genetic assay as well as three novel microsatellite markers that distinguish the two most common lineages found on the Great Barrier Reef. The assay is based on PCR amplification of two regions within the mitochondrial putative control region, which show consistent and easily identifiable fragment size differences for the two genetic lineages after *Alu1* restriction enzyme digestion of the amplicons.

## Introduction

Many scleractinian coral species show high morphological variability that has been related to differing environmental conditions. Reciprocal transplant experiments have demonstrated that the same genet may exhibit different growth forms in response to light and water movement intensities [1], [2]. However, some putative eco-morphs within single species represent distinct genetic lineages [3]–[9].

*Pocillopora damicornis* (Linnaeus, 1758) is a key model scleractinian coral species displaying a wide spectrum of morphotypes throughout its range [10], some of which occur in sympatry. The biology of this species, especially its reproductive modes, seasonality and lunar periodicity have often been found to be unexplainably variable, sometimes contradicting, among and even within studies, e.g. [11]–[15]. Recently, four independent studies have discovered sympatric, but seemingly reproductively isolated genetic lineages within the morphospecies *P. damicornis*: Flot et al. [16] report five distinct mitochondrial lineages from Hawaii, Pinzón and LaJeunesse [17] found three genetic lineages in the Tropical East Pacific, Souter [9] identified two from the coasts of Tanzania and Kenya, and Schmidt-Roach et al. [18] describe five lineages from the coast of Eastern Australia. The latter study showed that the genetic lineages of *P. damicornis sensu* [10] differ in their mode of reproduction, as well as in seasonal and lunar reproductive peaks. In light of these new results, the complex biology of the morphospecies *P. damicornis* should be re-visited, using the newly identified genetic lineages as the units of observation. Although the genetic lineages seem to be associated with phenotypic characteristics, phenotypic plasticity and cryptic appearance complicate confident identification, especially for the untrained eye. Currently, the only practical technique to differentiate among these genetic lineages is sequencing mitochondrial regions characteristic for each lineage [18]. However, the relatively high costs and time-consuming nature of this procedure make their routine application to large sample sets unrealistic.

This paper presents a quick and relatively low-cost genetic assay to reliably identify the two most common, and best resolved genetic lineages of *P. damicornis* from the Great Barrier Reef (GBR), Types α and β [18]. Our assay targets the mtDNA putative control region, as described in [19], by RFLP analysis of PCR amplicons. We tested the performance of the assay by comparing its results against two datasets: (i) 145 coral samples with known lineage identity based on a multi-locus (both nuclear and mtDNA) sequence analysis [18] (Table 1), and (ii) 329 samples of *P. damicornis sensu* [10], collected around Lizard Island, central GBR, and characterised by nine polymorphic microsatellite loci, three of which were developed as part of this study.

**Table 1:** Location and phylogenetic identities of *Pocillopora damicornis* samples from Schmidt-Roach et al. [18].

Location	Outgroup	Type α	Type β	Type γ	*Pocillopora verrucosa*	Type δ	Type ε	Total
Great Detached Reef			3	1	1		1	**6**
Great Keppel Island		19						**19**
Long Reef			3	1	2			**6**
Lord Howe Island		1+4*						**5**
Myrmidon Reef	1		1					**2**
Night Reef		3	3					**6**
Orpheus/Pelorus Island		3	19	4	10			**36**
One Tree Island		6						**6**
Rib Reef		2	3	1				**6**
Solitary Islands						3		**3**
Tydeman Reef			3		3			**6**
Wallace Islet Reef		21	3					**24**
Wilkie Reef		2	16	2				**20**
**Total**	**1**	**61**	**54**	**9**	**16**	**3**	**1**	**145**

All samples were correctly identified by the RFLP PCR assay as Type α, Type β or “other *Pocillopora*”. “Type α LHI” specimens (indicated with an *) were identified as “other *Pocillopora*” by the assay.

## Materials and Methods

### Ethics Statement

All necessary permits were obtained for the described field studies. Specimens for this study were collected under permit numbers G08/28215.1 and G09/30237.1, issued by the Australian Government’s Great Barrier Reef Marine Park Authority. The locations of sample collection are not privately-owned, and no endangered or protected species were collected.

### Assay Development

**Figure 1:**
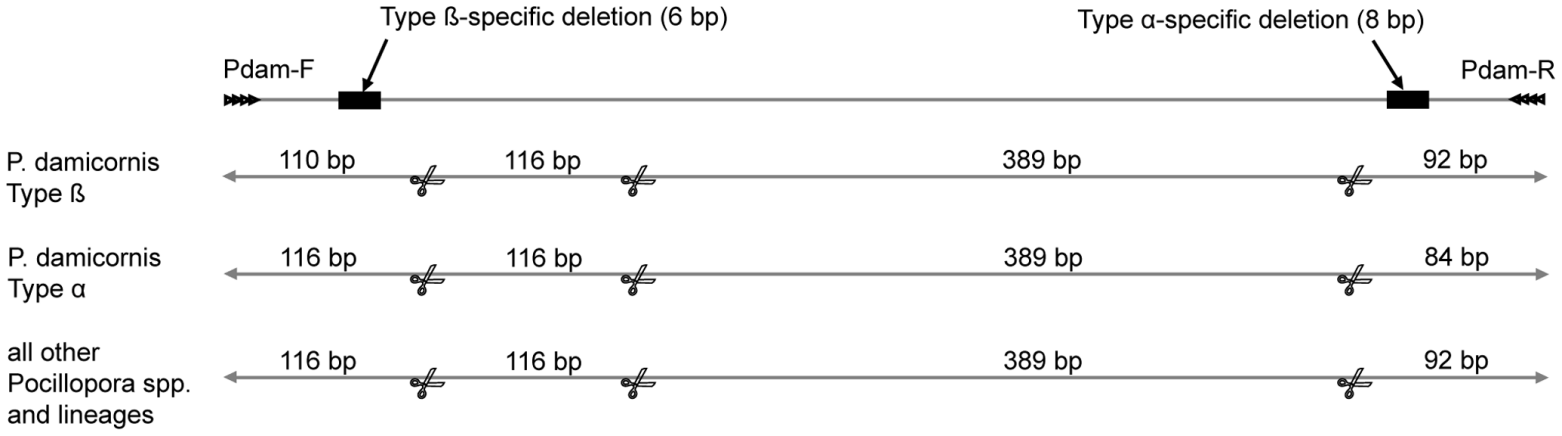
Schematic overview of the diagnostic mitochondrial putative control region. The primer pair Pdam -F and -R amplifies a region that contains two type-specific deletions: a 6-bp deletion in the mtDNA of Type β specimens, and an 8-bp deletion in Type α specimens. The amplicon, when subjected to *Alu1* restriction digestion, gives unique and readily recognizable fragment sizes for Type α, Type β and all other *Pocillopora* species and lineage types. Scissors indicate *Alu1* recognition sites, numbers show the size of the corresponding fragments in bp.

We searched the genome of pocilloporid species for conserved lineage-specific indels. Following unsuccessful trials on nuclear genomes, we aligned the putative control region (i.e., the region between the *atp8* and *cox1* genes [19] of the mitochondrial genome) of pocilloporid species, including the newly identified genetic lineages that are present on the GBR. The alignment of GenBank sequences NC_009797.1, NC_009798.1, NC_010244.2, NC_010245.2, NC_011162.1, NC_011160.1, JX624790– JX625114 in BioEdit v7.0.1 [20] using ClustalW Multiple Alignment [21] showed that an eight-bp deletion is characteristic for *P. damicornis* Type α and a six-bp deletion is unique for Type β, *sensu* Schmidt-Roach et al. [18]. The design of primers that directly bind to these lineage-specific indels was unsuccessful, therefore, using the web-based program Primer3 [22] we developed primers that target the region containing both indels, with a total length of 705 (Type α), 707 (Type β), and 713 bp (all other *Pocillopora* spp. and genetic lineages, hereafter “other *Pocillopora*”). Primer sequences are Pdam-F 5′-AAG AAG ATT CGG GCT CGT TT-3′ and Pdam-R 5′-CGC CTC CTC TAC CAA GAC AG-3′. These primer sequences do not occur in the mtDNA genome of the pocilloporid genera *Seriatopora* and *Stylophora*. The detection of such small amplicon size differences is challenging. Therefore, to facilitate rapid and reliable identification, i.e. to enable the use of simple agarose gels with reasonable electrophoresis time without compromising identification accuracy, we included a digestion step with *Alu1*. This restriction enzyme digest generates a unique banding pattern for Type α (84, 116 and 389 bp fragments), Type β (92, 110, 116 and 389 bp fragments) and “other *Pocillopora*” (92, 116 and 389 bp fragments; Figure 1), that is recognisable on a high density agarose gel.

### Testing the Assay

The assay was first tested on the 145 DNA samples that were used in the phylogenetic study of Schmidt-Roach et al. [18] (Table 1). PCR reactions were run in 10 µl volumes using a Qiagen Multiplex PCR Kit (Qiagen®), following the manufacturer’s protocol, with 2 pmol of both primers, and approximately 40 ng of the template DNA. Thermal cycling protocol was 15 min at 95°C, 30× (30 s at 95°C; 90 s at 60°C and 60 s at 72°C) and 10 min at 60°C. PCR products were subjected to Alu1 restriction enzyme digestion without purification for 1 hour at 37°C, using 5 µl of PCR product with 0.25 µl (2.5 U) of Alu1 enzyme and 1 µl of 10× NE Buffer 4 (BioLabs Inc.) in a 10 µl reaction. Five µl of the digested products were run on 4% TBE agarose gels (15×20.5 cm in size) for 4 hrs at 150 Volts. Banding patterns were scored manually. The resulting lineage identities of samples were compared to the published phylogenetic memberships [18].

We also compared the assay’s performance to an independent 329 *P. damicornis sensu* [10] multilocus microsatellite genotypes. These samples were collected at Lizard Island, northern GBR, and stored in absolute ethanol, until extracting DNA by a modified protocol of the salt precipitation method described in Wilson et al. [23]. Microsatellite primers developed by Magalon et al. [24] and Starger et al. [25] were tested for amplification and polymorphism on a random subset of 50 samples. One marker from Magalon et al. [24] and five from Starger et al. [25] were found to be polymorphic in our samples. To increase the discriminative power of genotyping, we developed additional microsatellite markers, as follows. Twelve *P. damicornis* colonies were collected at Orpheus Island, central GBR, and were transferred into aquaria at the Australian Institute of Marine Science. After an acclimatization time of four days, colonies were bleached with 10 µg l^−1^ of the herbicide diuron [26] over two weeks to remove most *Symbiodinium* cells from the coral tissue. Bleached fragments of each colony were preserved in absolute ethanol. DNA was extracted using the Qiagen DNEasy Tissue Kit and checked for *Symbiodinium* contamination by amplification of the ITS region, according to Coleman et al. [27]. None of the twelve samples showed amplification for *Symbiodinium* and were used for microsatellite isolation following Glenn and Schable [28]. After the creation of an enriched microsatellite library, primers for 15 microsatellites were designed using Primer3, and these loci were tested for polymorphism. Three marker sets were identified to amplify polymorphic microsatellite regions (Table 2), and were pooled with the selected markers from Magalon et al. [24] and Starger et al. [25] in three multiplex and one simplex PCR reactions (Table 3).

**Table 2:** Characteristics of newly developed microsatellite primers.

Locus	F sequence	R sequence	Motif	Amplicon size range (bp)	Tm
Pd4	ACGCACACAAACCAACAAAC	TAATTCCATCAACTCAAAGGGG	(AAAC)5	130-190	60°C
Pd11	TCGTTTGAAGGGAAATGCTC	GGCATGCTATGTATGCGAGA	(CA)7 T (AC)13	120-180	60°C
Pd13	TGTTCCTCTCTTTCTCTCTTCCA	CATTTATGTTCCTTTCACGGC	(TCTT)5	130-194	60°C

**Table 3:** Multiplex groups for microsatellite genotyping. A universal TET-labeled M13-F was added to groups M1 and M2.

Group	Locus	5' MOD	Meansize	runningTm	Source
M1	Pd3_004	TET (M13)	180	53°C	Starger et al. 2008
	PV7	HEX	220		Magalon et al. 2004
M2	Pd4	TET (M13)	148	60°C	new primer
	Pd11	FAM	157		new primer
	Pd3_002	HEX	200		Starger et al. 2008
M3	Pd13	HEX	153	60°C	new primer
	Pd3_008	TET	180		Starger et al. 2008
	Pd2_007	FAM	250		Starger et al. 2008
S	Pd3_009	FAM	350	52°C	Starger et al. 2008

All 329 *P. damicornis* DNA samples were genotyped at the nine microsatellite loci, following the Qiagen Multiplex PCR Kit (Qiagen®) protocol, using 0.5 pmol of the M13-tailed F primers, and 2 pmols of all other primers (Table 3). Thermal cycling was similar to the assay’s protocol, adjusting the annealing temperatures as per Table 3. PCR products were separated on a MegaBACE 1000 DNA Analysis System. Electropherograms were analyzed using MegaBACE Fragment Profiler v1.2 (Amersham Biosciences). All automatic scoring was checked manually.

The Bayesian clustering method implemented in STRUCTURE v2.3.3 [29] was run on the multilocus genotype dataset for K = 3, using the admixture model without locprior, and independent allele frequencies without initial population information, with a burn-in of 100,000 and 100,000 MCMC replications after the burn-in. Additionally, a Factorial Correspondence Analysis (FCA) was carried out in Genetix 4.05.2 [30] on the multilocus genotypes. The first two coordinates were plotted for visual analysis. The PCR RFLP assay was run on the same 329 DNA samples.

## Results and Discussion

**Figure 2:**
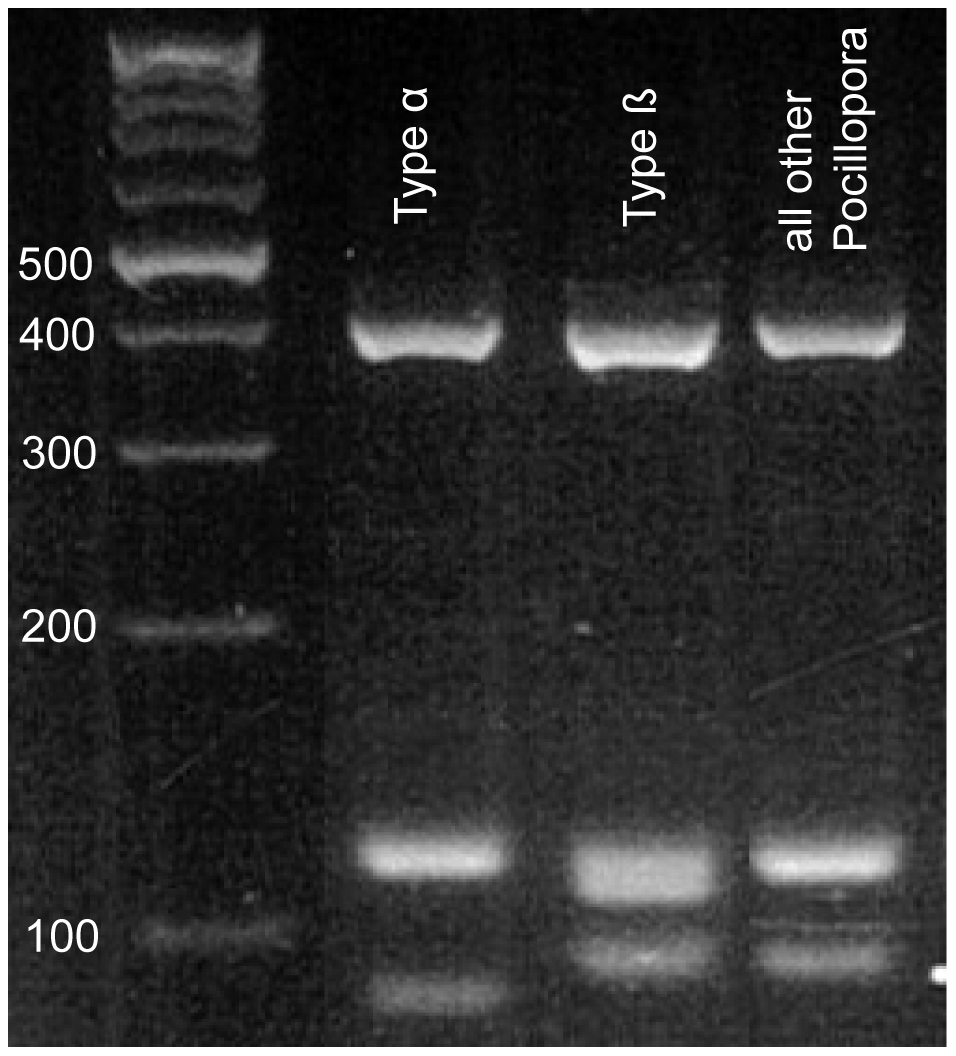
Image of 4% TBE agarose gel of the PCR RFLP assay products. A 100 bp ladder is run in the left-hand side lane.

Consistent with our expectations, three easily distinguishable banding patterns were seen on the agarose gels after running the assay on any *Pocillopora* DNA sample (Figure 2). The assay correctly identified all GBR samples from the study of Schmidt-Roach et al. [18] as either Type α, β or “other *Pocillopora*”, and classified the Type α LHI samples as “other *Pocillopora*” (Table 1). The taxonomic status of Type α LHI specimens is uncertain, but some evidence shows that they represent a distinct genetic lineage [18], therefore the assay correctly identified these as “other *Pocillopora*”. Since the GBR samples originate from 10 different reefs, spanning more than 12° of latitude, these results provide high confidence of the appropriateness of the assay as an identification tool for the whole of the GBR. Testing the assay on populations outside the East coast of Australia was beyond the scope of this study, therefore we recommend sequencing a representative number of specimens in any non-GBR population to determine whether the type-specific deletions are present, prior to adopting the presented assay as an identification tool.

**Figure 3:**
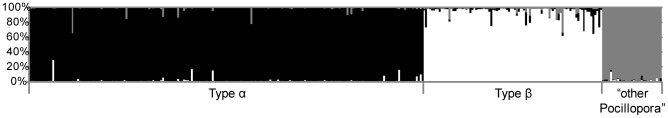
STRUCTURE plot (K = 3) of 329 *Pocillopora damicornis sensu* [**10**] specimens from Lizard Island. Genetic lineage was determined by the PCR RFLP assay.

**Figure 4:**
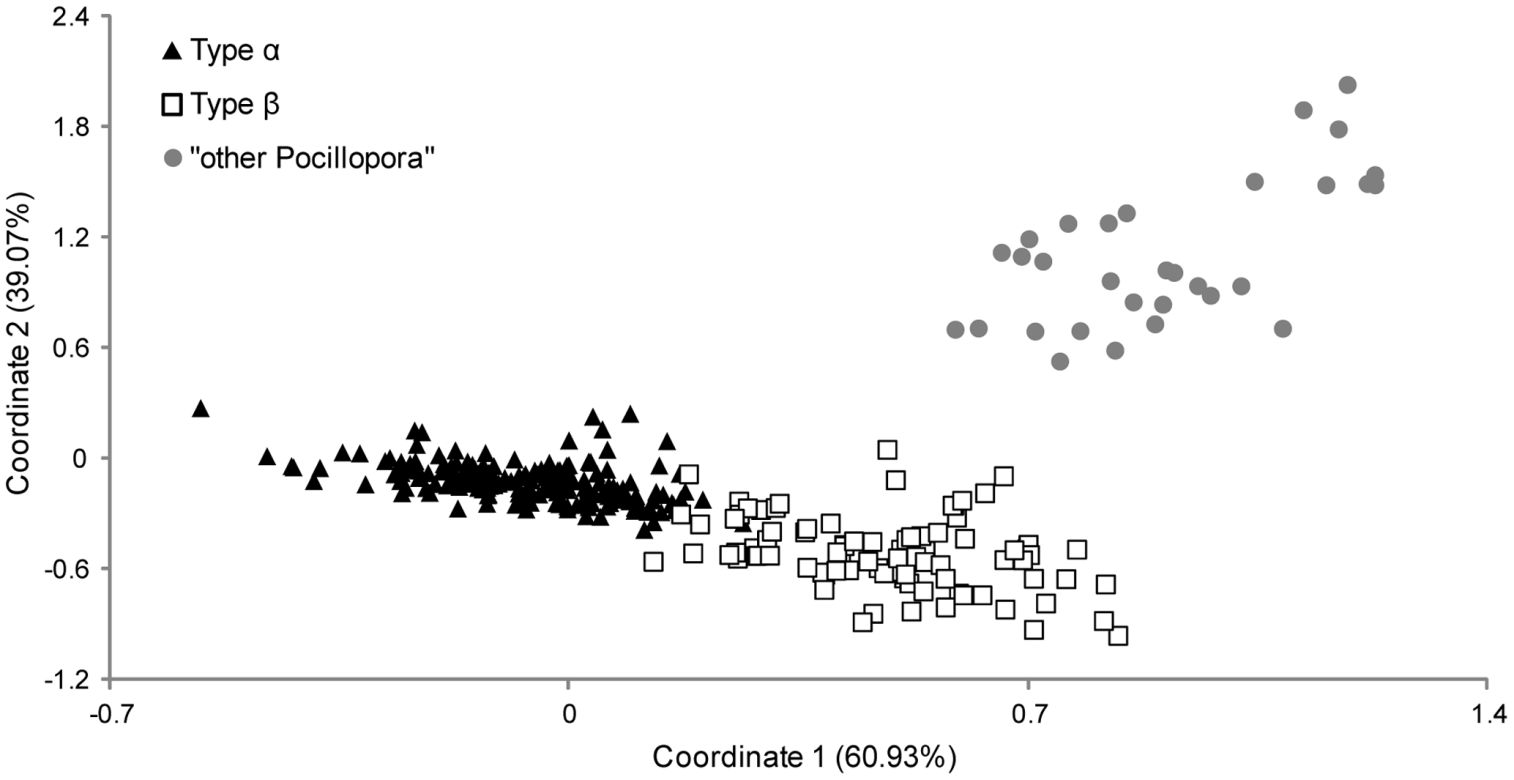
Results of the Factorial Correspondence Analysis on 329 *Pocillopora damicornis sensu* [**10**] microsatellite genotypes. The first two coordinates explain 100% of the variability. Genetic lineage was determined by the PCR RFLP assay.

The PCR RFLP assay identified 205 Type α, 93 Type β, and 31 “other *Pocillopora*” specimens among the 329 samples from Lizard Island. The Bayesian model clustered specimens according to their mtDNA lineage identity (Figure 3). Similarly, the FCA of the multilocus microsatellite genotypes showed three well distinguished clusters that correspond to the genetic lineage identity, assigned by the assay (Figure 4). The congruence between the nuclear microsatellite and mtDNA data strengthens the hypothesis that these lineages are genetically and hence reproductively isolated.

The genetic assay presented here provides a simple means to ensure that future studies avoid sample misidentification in *P. damicornis*. Furthermore, while the recruits of pocilloporid species are readily distinguished from the recruits of other scleractinian families by their characteristic skeletal structures [31], species identification of recruits merely by morphology is very unreliable. The primers of this assay were designed to only amplify *Pocillopora* spp. DNA, and not the DNA from other pocilloporid genera. The assay is therefore also suitable to reliably identify Type α and Type β *P. damicornis* from among pocilloporid recruits, which will open doors to genetic lineage-specific recruitment studies.
